# Responses of plant species diversity and soil physical-chemical-microbial properties to *Phragmites australis* invasion along a density gradient

**DOI:** 10.1038/s41598-017-11205-0

**Published:** 2017-09-08

**Authors:** MD Nazim Uddin, Randall William Robinson

**Affiliations:** 1 Department of Ecology and Environmental Management, College of Engineering & Science, Victoria University, St Albans Campus, Melbourne, Vic. 8001 Australia; 2 Institute of Sustainability & Innovation, College of Engineering & Science, Victoria University, Melbourne, Vic. 8001 Australia

## Abstract

The invasion of ecosystems by strongly colonising plants such as *Phragmites australis* is viewed as one of the greatest threats to plant diversity and soil properties. This study compared a range of diversity measures including soil properties and mycorrhizal potential under different degrees of *Phragmites* density among three populations in coastal wetland, Victoria, Australia. Species richness, evenness and Shanon-Wiener index had significantly higher values in low degree of *Phragmites* density in all populations. Higher densities had the lowest diversity, with Shannon-Wiener index = 0 and Simpson’s index = 1 indicating its mono-specificity. Significant alterations in soil properties associated with different degrees of *Phragmites* density were noticed. These had interactive effects (population × density) on water content, dehydrogenase activity, microbial biomass (C, N and P) but not on pH, electrical conductivity, phenolics, organic carbon, and spore density. Furthermore, the study elucidated decrease of competitive abilities of native plants, by interfering with formation of mycorrhizal associations and biomass. Overall, our results suggest that significant ecological alterations in vegetation and soil variables (including mycorrhizal potential) were strongly dependent on *Phragmites* density. Such changes may lead to an important role in process of *Phragmites* invasion through disruption of functional relationships amongst those variables.

## Introduction

Biological invasions by strongly competitive native and non-native species, (commonly referred to as invasive species), is a worldwide phenomenon that threatens to dramatically change communities and ecosystems^1, 2^. The expansion of invasive plant species both with and without human influence, has caused striking modifications of natural ecosystems that threaten critical ecosystem services^3^. Invasive plants are characterized by high productivity associated with several key competitive mechanisms that assist invasion, including greater nutrient use efficiency, early maturation, and allelopathy^4^. Many invasive species are tolerant of or even adapted to environmental disturbance, which is likely to accelerate their expansion^5^. As a result, they often displace less competitive native species, resulting in the loss of plant diversity^1^, which in turn, weakens the stability and functioning of ecosystems^6^.

Wetlands, including brackish marshes, represent some of the most productive natural ecosystems. They support various biological communities and biogeochemical functions providing heterogeneous nutrient conditions and diverse habitats, and they are key drivers of global nutrient cycling^7^. Topographic features of wetlands, such as their location in natural depressions and their frequent connectivity to both terrestrial and aquatic systems, make these ecosystems vulnerable to anthropogenic disturbances^8^. These features can facilitate the invasion of both non-native and native plants.

*Phragmites australis* (hence after *Phragmites*) is one of the most aggressive and widely studied invasive plant globally both within its native range and in areas where it is introduced^9^. The exact native range of *Phragmites* is obscure^10^, but it is considered to be native to large parts of the world and may even have the widest distribution of any flowering plant. Though it has been considered as native in some parts of Australia, it is regarded as environmental weed (invasive native) due to its invasive characteristics into ecosystems^11^. Highly adaptable, with great competitive ability, *Phragmites* can grow in a wide variety of ecosystems and plant communities including wetlands, coastal marshes, inland lakes and rivers, mountains, deserts, and urban areas^12^. *Phragmites* can form monocultures that dominate in the ecosystem for longer time scales than other plants by significantly modifying the native ecosystem structure and functions^13, 14^. Physiological characteristics of *Phragmites* including high rates of reproduction, well-developed aerenchyma and rhizosphere facilitate colonization. Dense canopies related to their high productivity can inhibit germination and growth of other plant species^15, 16^. Given the different tissue chemistry of *Phragmites* from other species in many native plant communities, its extensive invasion can alter nutrient pools and nutrient availability in soils^15^. Additionally, greater biomass production and differential decomposition rates potentially cause changes in organic matter accumulation that, in turn may lead to further topographic and hydrologic changes^17^. Since many ecosystem functions are mediated by soil microbial communities, ecosystem-level changes in wetland biogeochemistry resulting from the spread of invasive plants may stimulate either positive and negative microbial responses^18, 19^. On the other hand, some studies have reported that plant invasions may supply additional organic matter and oxygen to soil and thereby, increase denitrification and enzyme (especially phosphatase) activities^20, 21^. Contrasting responses of microbial biomass and community structure to plant invasion have also been observed^19, 22^.

Despite the growing understanding of the biology of invaders, and increasing efforts to restore invaded landscapes, far less attention has focused on the consequences of plant invasions for the communities they invade, particularly the effects by different degrees of plant invasion^23, 24^. Invasive plant species are often assumed to alter plant species richness, diversity, and/or composition, as well as soil properties including microbial activities, but yet, only a handful of studies have quantitatively evaluated such possibilities at a community level, with no studies for *Phragmites* in Australia undertaken to date. Therefore, in this study, we examined how different degrees of invasion of a former Chaffy Saw Sedge (*Gahnia filum)* and Swamp Paperbark (*Melaleuca ericifolia*) coastal wetland dominated by *Phragmites* would affect community plant diversity and soil properties, including microbial with mycorrhizal potential.

## Results

### Impact on diversity in plant communities

**Figure 1:**
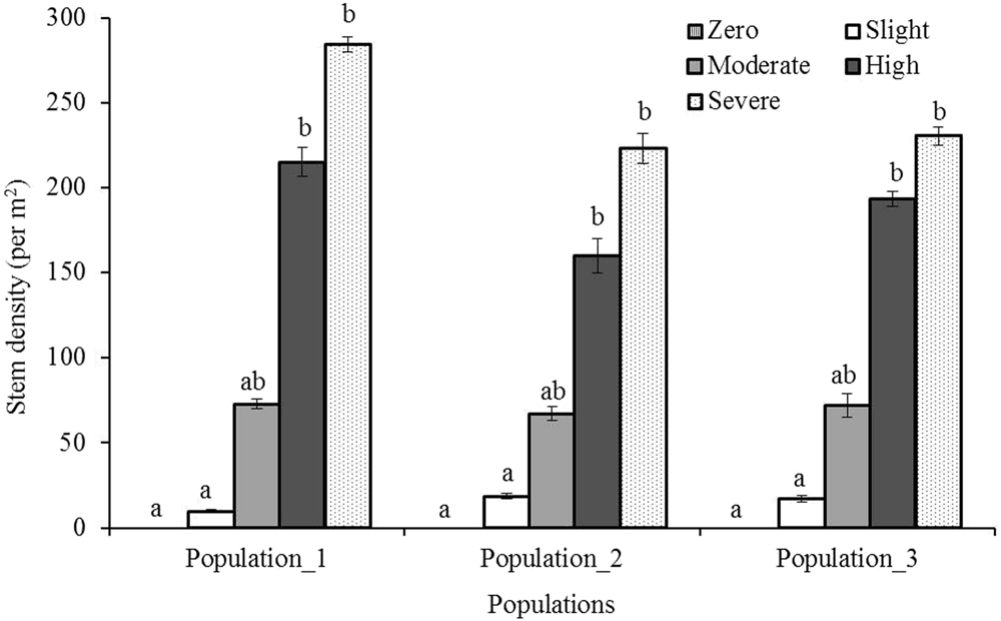
Stem density (per square meter) of live *Phragmites australis* along density gradient in three populations. Data are mean ± SE, *n* = 3. Different lower case letters over the bar indicate significant differences amongst density categories within each population (*P* ≤ 0.05).

**Figure 2:**
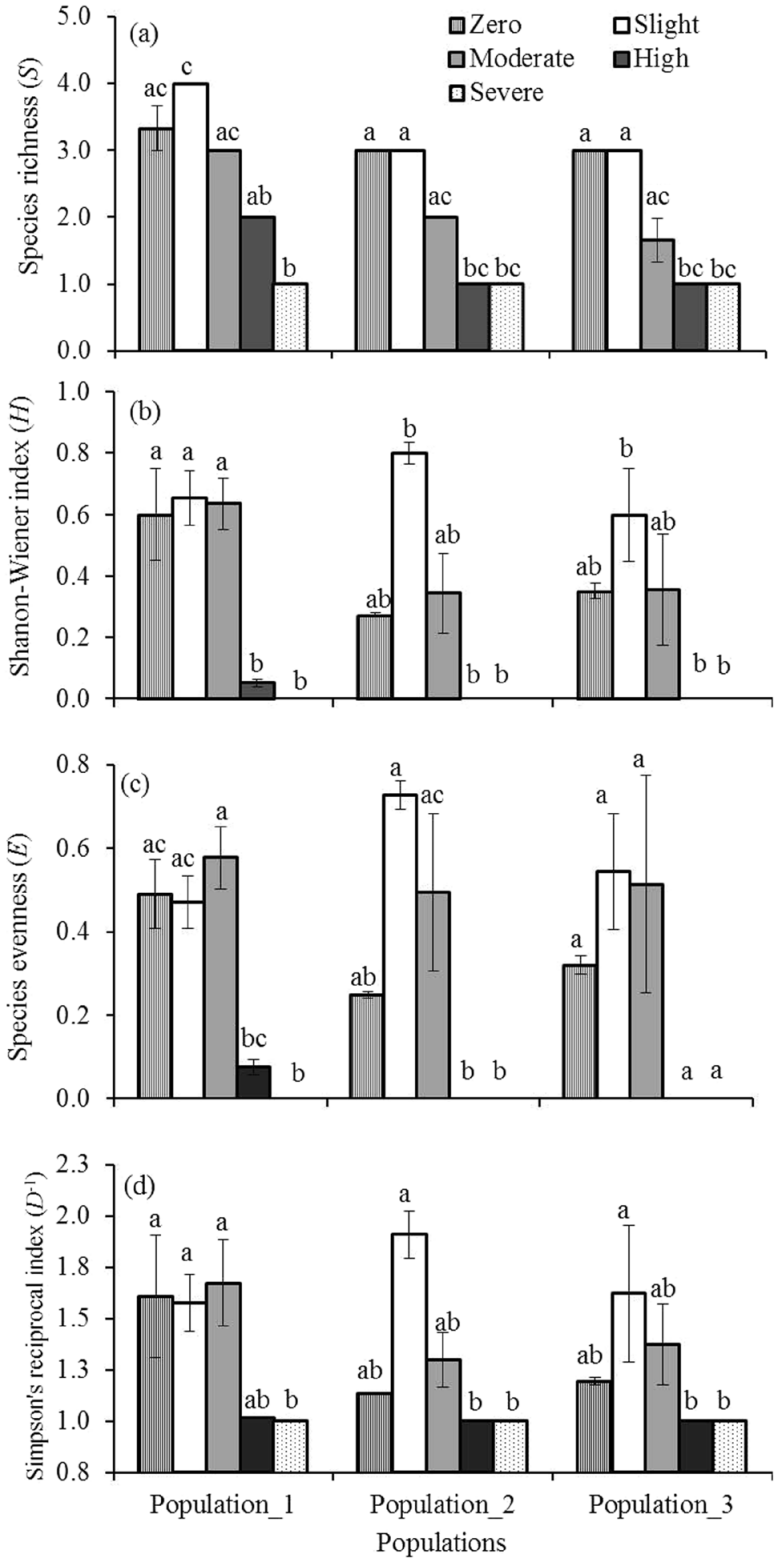
Density-dependent impact of *Phragmites australis* on (**a**) species richness (*S*), (**b**) Shanon-Wiener Index (*H*), (**c**) species evenness (*E*) and (**d**) Simpson’s reciprocal index (*D* ^*-1*^) in three populations. Data are mean ± SE, *n* = 3. Different lower case letters over the bar indicate significant differences amongst density categories within each population (*P* ≤ 0.05).

Along the transects, the density of *Phragmites* shoots increased from none to maximal values over a distance of only 12 m (Fig. 1) and the density was significantly varied among invasion categories within each population (Kruskall-Wallis: *χ* ^2^ = 13.62, 13.59 & 13.54 with df = 4 and p ≤ 0.05 for population 1, 2 & 3 respectively). The maximum density (285) was found in population_1 compared to others but there was no significant difference among the populations (ANOVA: *F*_2,42_ = 0.212, p ≥ 0.05). A total of 9 plant species was identified in the random quadrats of the three populations (Table 1). The number of species per quadrat was on average highest in the low density *Phragmites* stand and had been lowering as *Phragmites* density increased (Fig. 2). The diversity indices: species richness (*S*), Shannon-Wiener diversity index (*H)*, species evenness (*E*) and Simpson’s reciprocal index (*D* ^*−*1^) differed significantly among the density categories within each population (For example, in population_1: Kruskall-Wallis: *χ* ^2^ = 13.27, 11.18, 11.28 & 11.01 with df = 4 and p ≤ 0.05 for *S*, *H*, *E* & *D* ^*−*1^ respectively) but there were no significant differences among three populations (ANOVA: *F*_2,42_ = 2.45, 0.709, 0.095 & 0.550, p ≤ 0.05 for *S*, *H*, *E* & *D* ^*−*1^ respectively). Values of all indices were significantly higher in low density compared to severe density in all populations. (Fig. 2: Dunn-Bonferroni, *p* < 0.05 for all pairwise comparisons). In all populations, the severe density had the lowest diversity, with *H* = 0 and *D* ^*−*1^ = 1, i.e. they were monospecific stands. Again, the regression analysis showed that *Phragmites* stem density could significantly predict all the indices of the diversity in the community (For example, in population_1: *F*_1,13_ = 112.25, 48.78, 43.80 & 14.38, *P* ≤ 0.002; R^2^ = 0.89, 0.79, 0.77 & 0.53 for *S*, *H*, *E* & *D* ^*−*1^ respectively). Together these results indicate that *Phragmites* dominated the marshes and suppressed other plant species.

**Table 1:** A total of identified species in the invaded zone of three populations.

Name of species	Population_1	Population_2	Population_3
*Rumex conglomeratus*	√	√	√
*Atriplex hastata*	√	√	√
*Eleocharis acuta*	√	√	√
*Bolboschoenus caldwellii*	√	√	√
*Phragmites australis*	√	√	√
*Rumex spp*	√	√	√
*Pennisetum clandestinum*	√	×	×
*Sonchus aspera*	×	√	×
*Brassica napus*	×	√	×

### Impact on soil properties

**Figure 3:**
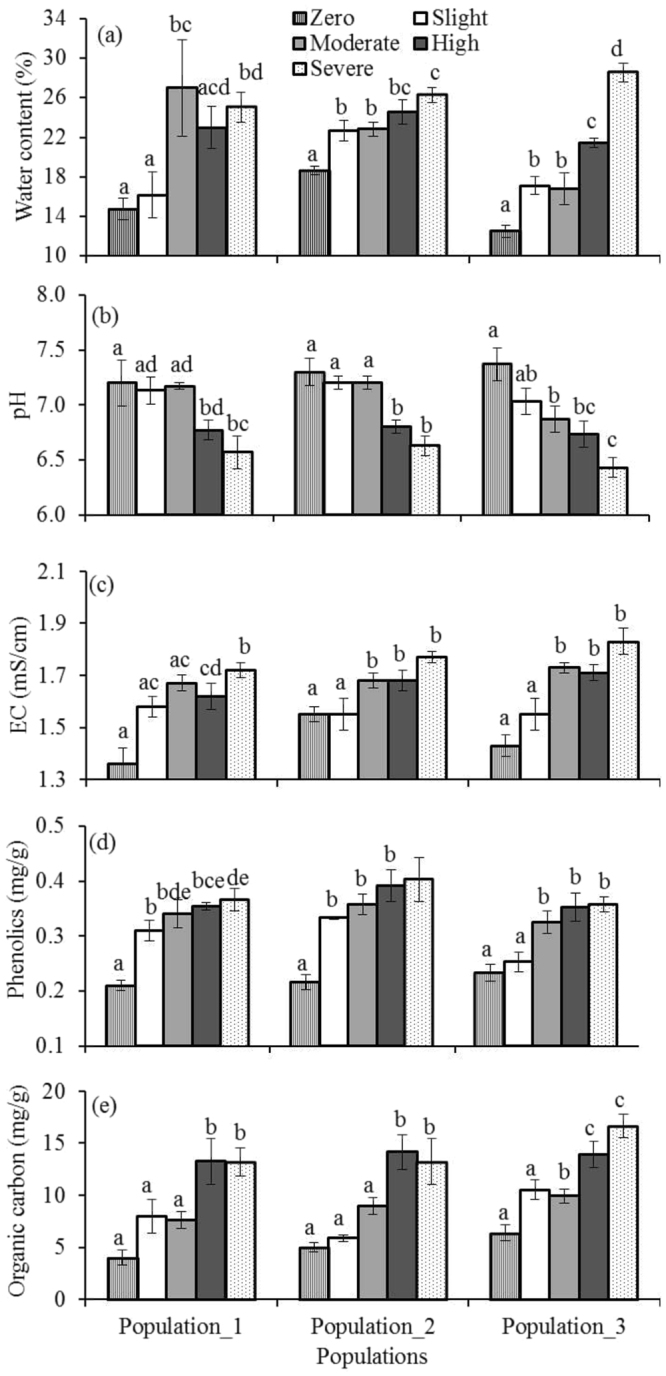
Density-dependent changes of *Phragmites australis* on soil physico-chemical properties (**a**) water content, (**b**) pH, (**c**) EC, (**d**) phenolics and (**e**) organic carbon in three populations. Data are mean ± SE, *n* = 3. Different lower case letters over the bar indicate significant differences amongst density categories within each population (*P* ≤ 0.05).

**Figure 4:**
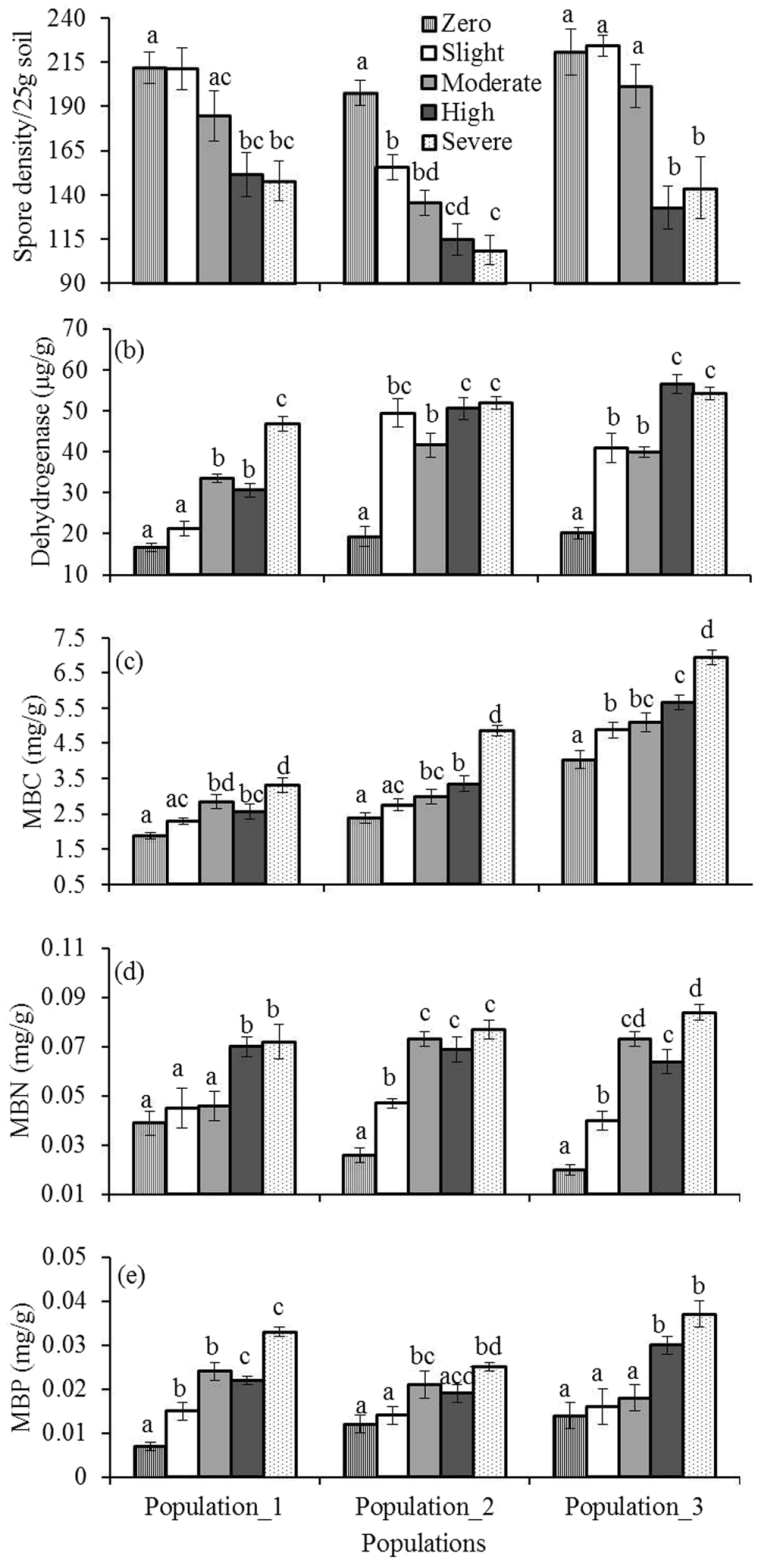
Influence of *Phragmites australis* density on biological properties of soil (**a**) arbuscular mycorrhizal fungi (AMF) spore density, (**b**) dehydrogenase activity (DHA), (**c**) microbial biomass carbon (MBC), (**d**) microbial biomass nitrogen (MBN), and (**e**) microbial biomass phosphorous (MBP) in three populations. Data are mean ± SE, *n* = 3. Different lower case letters over the bar indicate significant differences amongst density categories within each population (*P* ≤ 0.05).

ANOVA results revealed that the degrees of *Phragmites* density × population interactions had significant effects on soil properties: water content (WC), dehydrogenase activity (DHA), microbial biomass (C, N and P), except pH, electrical conductivity (EC), phenolics, organic carbon (OC), and spore density (SD) (Table 2). Relatively higher and statistically significant WC, EC, phenolic contents, OC with lower pH were found in highly density sites than in other density categories in all populations (Fig. 3). For example, in population_1, the physico-chemical properties like WC (F_4,10_ = 3.98, *P* < 0.03), pH (F_4,10_ = 4.58, *P* < 0.02), EC (F_4,10_ = 9.25, *P* < 0.002), phenolics (F_4,10_ = 12.75, *P* < 0.001), and OC (F_4,10_ = 7.55, *P* < 0.005) varied significantly across the density categories. Significantly higher DHA, microbial biomass C, N and P with lower AMF spore density were found in highly density categories than in others (Fig. 4). Biological properties like AMF spore density (F_4,10_ = 6.88, *P* < 0.006), DHA (F_4,10_ = 62.04, *P* < 0.001), MBC (F_4,10_ = 10.61, *P* < 0.001), MBN (F_4,10_ = 6.76, *P* < 0.007), and MBP (F_4,10_ = 25.14, *P* < 0.001) varied significantly across the density categories, for example, in population_1. Linear regression indicated that density status significantly influenced the soil properties in all populations. For example, in population_1: the relationships were like with WC (*F*_1,13_ = 5.33, *P* ≤ 0.038; R^2^ = 0.29), pH (*F*_1,13_ = 20.97, *P* ≤ 0.001; R^2^ = 0.61), EC (*F*_1,13_ = 8.95, *P* ≤ 0.01; R^2^ = 0.41), phenolics (*F*_1,13_ = 11.48, *P* ≤ 0.005; R^2^ = 0.47), OC (*F*_1,13_ = 24.80, *P* ≤ 0.001; R^2^ = 0.65), SD (*F*_1,13_ = 26.34, *P* ≤ 0.001; R^2^ = 0.67), DHA (*F*_1,13_ = 35.14, *P* ≤ 0.001; R^2^ = 0.73), MBC (*F*_1,13_ = 14.00, *P* ≤ 0.002; R^2^ = 0.51), MBN (*F*_1,13_ = 30.83, *P* ≤ 0.001; R^2^ = 0.70), and MBP (*F*_1,13_ = 22.79, *P* ≤ 0.001; R^2^ = 0.63).

**Table 2:** Results of two-way ANOVA (*F*-ratios) evaluating the density-dependent role of *Phragmites australis* on soil properties.

Factor	*df*	*F* ratio and probability									
		WC	pH	EC	Phenolic	OC	SD	DHA	MBC	MBN	MBP
Populations (P)	2	5.66**	1.93 ^ns^	3.29 ^ns^	4.07*	5.97**	22.71***	57.78***	270.32***	0.85 ^ns^	4.85*
Density categories (D)	4	18.22***	21.09***	29.05***	27.72***	32.94***	31.6***	97.17***	56.50***	58.45***	33.28***
P × D	8	2.97*	0.67 ^ns^	1.44 ^ns^	0.9 ^ns^	0.88 ^ns^	1.41 ^ns^	8.63***	3.14**	4.44**	3.18**
Error	30										
Total	45										

WC, water content; EC, electrical conductivity; OC, organic carbon; SD, spore density; DHA, dehydrogenase activity; MBC, microbial biomass carbon; MBN, microbial biomass nitrogen; and MBP, microbial biomass phosphorous. Level of significance: ***, **, * and ^ns^ indicate significant difference at *P* ≤ 0.001, *P* ≤ 0.01, *P* ≤ 0.05 and non-significant respectively.

### Correlations and PCA

**Figure 5:**
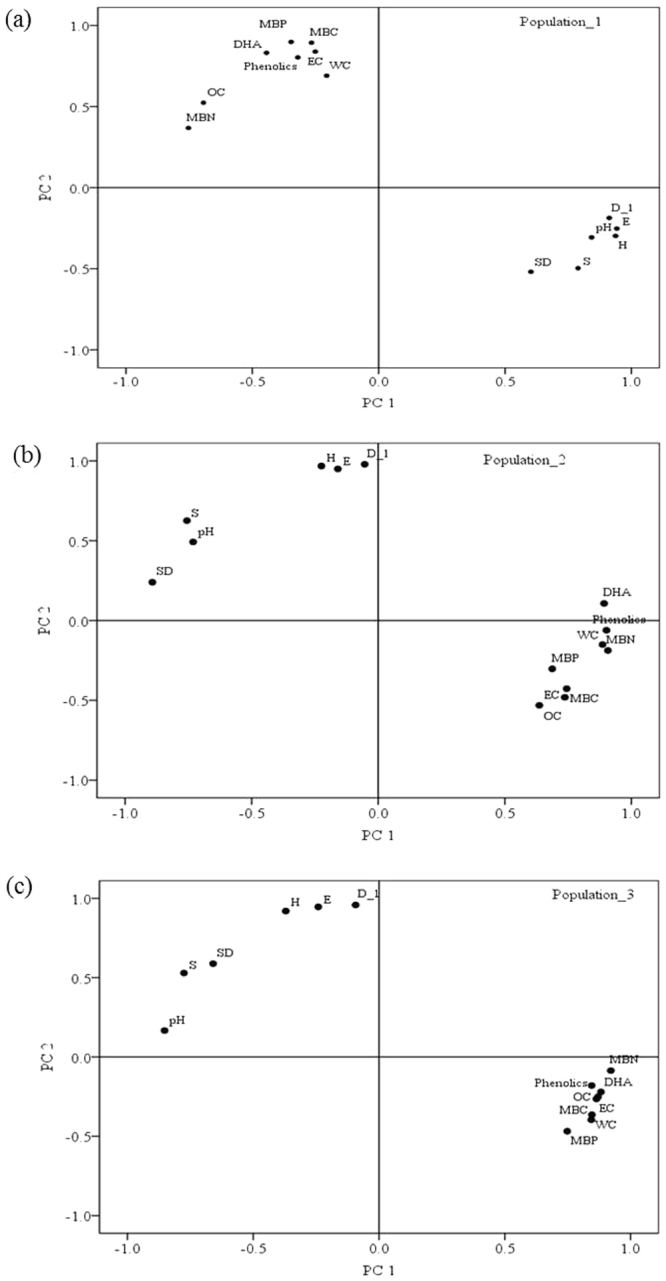
Principal-components analysis (PCA) loadings of diversity indices and soil properties (physical-chemical-biological) from three populations (**a**) population_1, (**b**) population_2, and (**c**) population_3. Biodiversity indices: *H*, Shannon-Wiener diversity; *S*, species richness; *E*, species evenness; and *D* ^−1^, Simpson’s reciprocal index. Soil properties: WC, water content; EC, electrical conductivity; OC, organic carbon; SD, spore density; DHA, dehydrogenase activity; MBC, microbial biomass carbon; MBN, microbial biomass nitrogen; and MBP, microbial biomass phosphorous.

Significant negative correlations were found among soil properties and biodiversity indices in each population, except pH and AMF spore density (Table 3). Within each site, PCA consistently revealed two components of variation among variables. The loadings on the two principal components were similar at all sites except population_1 (Fig. 5). In population_1, two principal components were identified with eigenvalues larger than 1, explaining approximately 81% of the total variance. The first component, which explains 68% of the total variance within the dataset, is characterized by high positive loadings for all biodiversity indices with soil pH and AMF spore density. The second component, which explains a further 13% of the variance, is characterized by high positive loadings for EC, phenolics, DHA, MBC and MBP. Whereas in population_2 and population_3, PC1 accounted for 65–70% of the variation (eigenvalues and R^2^, respectively, for: poulation_2: 9.03, 64.51; and poulation_3: 9.86, 70.44) and PC2 accounted for 14–18% of the variation (poulation_2: 2.49, 17.79; and poulation_3: 1.98, 14.15) within sites.

**Table 3:** Correlation coefficients between soil properties and biodiversity indices in each population invaded by *Phragmites australis*.

	Population_1				Population_2				Population_3			
	*H*	*S*	*E*	*D* ^*−1*^	*H*	*S*	*E*	*D* ^*−1*^	*H*	*S*	*E*	*D* ^*−1*^
WC	−0.44	−0.51*	−0.37	−0.43	−0.34	−0.75**	−0.30	−0.21	−0.62*	−0.75**	−0.57*	−0.41
pH	0.85**	0.81**	0.83**	0.81**	0.60*	0.84**	0.60*	0.47	0.49	0.76**	0.41	0.21
EC	−0.49	−0.53^*^	−0.46	−0.45	−0.63*	−0.78**	−0.56*	−0.51*	−0.57*	−0.82**	−0.41	−0.40
Phenolics	−0.51*	−0.53*	−0.50	−0.44	−0.27	−0.73**	−0.21	−0.11	−0.49	−0.80**	−0.32	−0.29
OC	−0.78**	−0.69**	−0.79**	−0.73**	−0.64**	−0.88**	−0.58*	−0.51	−0.53*	−0.74**	−0.49	−0.29
SD	0.68**	0.79**	0.64**	0.51*	−0.63*	−0.90**	−0.56*	−0.50	−0.51	−0.74**	−0.46	−0.25
DHA	−0.64**	−0.83**	−0.61*	−0.50	0.44	0.85**	0.38	0.27	0.77**	0.84**	0.71**	0.54*
MBC	−0.53*	−0.69**	−0.49	−0.40	−0.11	−0.59*	−0.12	0.06	−0.50	−0.77**	−0.43	−0.28
MBN	−0.76**	−0.77**	−0.77**	−0.68**	−0.54*	−0.77**	−0.52*	−0.42	−0.65**	−0.79**	−0.59*	−0.46
MBP	−0.59*	−0.75**	−0.56**	−0.47	−0.42	−0.69**	−0.29	−0.37	−0.45	−0.83**	−0.27	−0.19

Soil properties: WC, water content; EC, electrical conductivity; OC, organic carbon; SD, spore density; DHA, dehydrogenase activity; MBC, microbial biomass carbon; MBN, microbial biomass nitrogen; and MBP, microbial biomass phosphorous. Biodiversity indices: H, Shannon-Wiener diversity; S, species richness; E, species evenness; and D^−1^, Simpson’s reciprocal index. ** and * indicate the significant at the 0.01 and 0.05 level respectively.

### Mycorrhizal inoculum potential of soil

**Figure 6:**
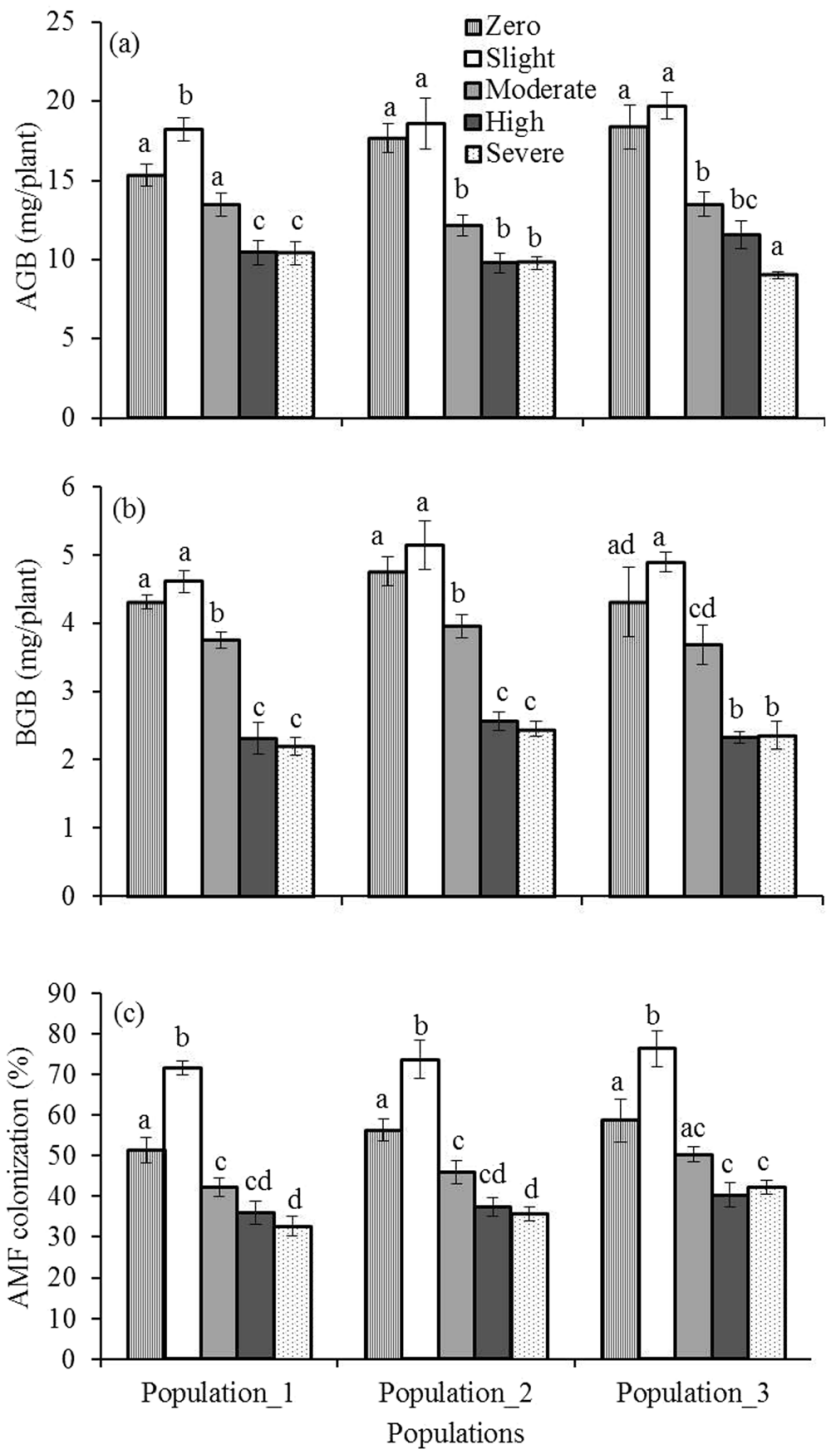
Influence of *Phragmites australis* invaded soil along density gradient on (**a**) above-ground biomass (AGB), (**b**) below-ground biomass (BGB), and (**c**) arbuscular mycorrhizal fungus (AMF) colonization of *melaleuca ericifolia* in three populations. Data are mean ± SE, *n = 3*. Different lower case letters over the bar indicate significant differences amongst density categories within each population (*P* ≤ 0.05).

The density status × population interactions had no significant effect on the growth and establishment of *Melaleuca ericifolia* like above-ground biomass (AGB) (*F*_8,30_ = 1.26, *P* ≥ 0.05), below-ground biomass (BGB) (*F*_8,30_ = 0.19, *P* ≥ 0.05), and AMF colonization (*F*_8,30_ = 0.18, *P* ≥ 0.05) but individually density status had significant effect on all parameters (*F*_4,30_ = 63.53, *P* ≤ 0.001 for AGB, *F*_4,30_ = 80.14, *P* ≤ 0.001 for BGB, and *F*_4,30_ = 74.74, *P* ≤ 0.001 for AMF colonization) as well as population had no significant effect on AGB (*F*_2,30_ = 1.58, *P* ≥ 0.05) and BGB (*F*_2,30_ = 3.05, *P* ≥ 0.05) except AMF colonization (*F*_2,30_ = 6.20, *P* ≤ 0.05). *Phragmites* infested soil also significantly reduced the AGB, BGB and AMF colonization in *Melaleuca ericifolia* across the density categories in all populations (Fig. 6).

## Discussion

A negative linear relationship between invasive species and species diversity in a given area is a general trend, which might be changed by a variety of environmental stressors. For example, soil moisture (water availability) may influence the relative performance of native and non-native invasive species. *Phragmites* can gain higher competitive advantage over other plant species (natives) in aquatic (highly moisturised) vs. terrestrial ecotypes^25, 26^. Similarly, natives have superior physiological performance with diversity compared with invasive under less soil moisture condition^27^. Considering these, our findings regarding the relationships between the cover of *Phragmites*/others and plant community diversity were well supported with other studies^26^ i.e. less cover of *Phragmites* had low soil resources (moisture and nutrients) with high biodiversity indices. Again, native species are often considered to be the driving force in increasing biodiversity at local scales^28^. In the present study, the native plants in the communities invaded by *Phragmites* contributed to increasing plant diversity when the density of *Phragmites* was at slight and moderate level. Once the cover exceeded, a negative effect of density on local species diversity appeared, possibly due to disequilibrium of communities caused by the rapid growth of invasive plants^29^.

We also found the examined biodiversity indices (species richness, species diversity, species dominance, and species evenness) did not linearly decrease/increase with increases of *Phragmites* density. Instead, *Phragmites* slightly increased the plant diversity of the invaded community within a certain range of density values (slight density phase), possibly due to direct or indirect facilitation of non-native species during their initial introduction^30^. For example, invasive *Sargassum muticum* increased native species richness at a low percent cover^31^. This finding is consistent with a study by Melo *et al*.^32^ indicating that human disturbance at an intensity below a certain threshold would not trigger irreversible biodiversity loss, and the delivery of ecosystem services would increase up to a point with increasing human disturbance. In this study, all the three populations were monospecific at high and severe density levels. However, *Phragmites* does not always lead to monospecific stands. If the area presents suboptimal conditions for *Phragmites*, such as fairly low water table or low nutrient availability, or if strong competitors are present, *Phragmites* may not be vigorous enough to competitively displace existing species and may coexist with them^33^. In this study, we observed that adjacent to the monospecific *Phragmites* stand in Cherry Lake, a zone of very tall and dense populations of the equally invasive *Typha orientalis* (or *Typha spp*.), overtopped the *Phragmites* and probably prevented it from becoming more dominant.

Despite numerous efforts to examine the influences of invasive plants on soil characteristics (physical, chemical and biological), inconsistent results are often obtained, especially under field conditions^17, 22^. These inconsistencies possibly arise from complex and interlinked factors such as large spatial and temporal variation in soil characteristics, and uneven plant diversity between undisturbed and invaded regions. In this study, we selected sites that were located within a short distance of one another, and represented areas of undisturbed topography where former plant diversity was more or less similar between presently invaded and uninvaded areas. We anticipated this approach would provide an improved understanding of the influence of invasive plants on the soil properties by differentiating effects of modified soil characteristics and loss of plant diversity by plant invasion.

In this study, different degrees of *Phragmites* density significantly influenced a range of soil physical-chemical-biological characteristics. High soil organic carbon, phenolics contents with low pH in invaded areas can be explained by additional organic matter supply by *Phragmites* through root exudation^11, 14, 34^ and litter fall associated with their high productivity^15^. Along with the changes in soil physical-chemical characteristics, different degrees of *Phragmites* density had differential effects on belowground microbial activities. We found *Phragmites* density accelerated DHA, an indicator of microbial activities, which was consistent with the findings that phenolics may increase microbial activity^35^. These results were well aligned in this study due to linear relationships among increased organic carbon, phenolics contents, and dehydrogenase activity in *Phragmites* rhizosphere as well other studies Saviozzi *et al*.^36^ stating that soil enzymatic activities are well related to organic carbon and phenolics..

We also found soil microbial biomass C, N and P were greatest underneath *Phragmites* and this increase relates to the degree of density substantiated with the study of Stefanowicz *et al*.^37^ Martina *et al*.^38^. Our results clearly showed that soil properties under the canopy of *Phragmites* were profoundly different from areas outside the canopy even if they were only located a few metres away. The degree of organic matter accumulation in soil is generally dependent on the relationship between C input and decomposition rates from litter of plants. As *Phragmites* is a perennial graminaceous plant it returns more C to the soil from dying leaves and roots, than annual plants in the surrounding community^39^.This was clearly linked to high organic carbon accumulation in highly density soil in this study. The higher organic carbon in *Phragmites* invaded soil presumably contained substrates such as sugars, amino acids and organic acids that were readily metabolized, leading to the greater concentration of microbial biomass. The findings are well justified with the results of Yang *et al*.^40^ through confirmation of positive soil microbial biomass carbon correlation with soil organic carbon.

The significant changes in microbial biomass and DHA were clearly associated with the different degrees of *Phragmites* density. The lower microbial biomass and DHA in the soil beneath the low degree density around patches of *Phragmites* may be caused by the absence of carbon inputs^37, 40^. From the present study, again, we found variations of AM spore abundance and inoculum potential in soil colonised by *Phragmites*, which is aligned with the study of Sanon *et al*.^41^ whom found lower spore density from invaded sites by the invasive species, *Amaranthus viridis*. Moreover, some studies have found that increased soil fertility shifted the microbial community structure and biomass, with a noticeable decrease in fungi and increase in bacteria^42^ thereby validated in our mycorrhizal study findings. Again, it is assumed that the changes within microbial population are expected to affect the microbial biomass C:N ratio^43^, reflected in our study.. More acidic soils found under high degree density of *Phragmites* compared to others also supports the hypothesis that *Phragmites* has a greater influence on the proportion of microbial biomass depending on density.

Densely populated *Phragmites* soils showed a lower AMF inoculum potential in terms of *Melaleuca ericifolia* roots colonized by AMF in field collected soils. These results are directly compatible with other studies, most notably in a study of garlic mustard infested soil. Roberts and Anderson^44^ reported a significant negative correlation between density of garlic mustard and inoculum potential of AMF. In addition, Stinson *et al*.^45^ found invasive species *Alliaria petiolata* supressesed the native plant growth by disrupting the mutualistic association of belowground AMF potential with native tree seedlings. The results presented here suggest that reduced growth of *Melaleuca ericifolia* in *Phragmites* infested soil was linked to the AMF potential in soil. These findings strongly suggest AMF potential could be characterised as indirect allelopathy of invasive species^45^, for example, *Phragmites*. However, other factors such as nutrient dynamics^17^ may alter plant growth and AMF composition and this complex interplay needs further investigation.

In conclusion, this study highlighted the variations of plant diversity indices, and soil properties including mycorrhizal inoculum potential influenced by different degrees of *Phragmites* density. Specifically, *Phragmites* depending on density greatly reduced the biodiversity indices and increased the DHA and soil microbial biomass (C, N, P). In addition, significant decrease of the mycorrhizal infection and AMF spore density were found at sites of the highest compared with lowest *Phragmites* density. Additionally, higher phenolic content, lower pH and mycorrhizal inoculum potential of soil in high density sites demonstrate that *Phragmites* competitive success may be partly achieved due to release of allelopathic compounds; a finding compatible with other studies^11, 14, 34, 46–48^. This study represents a step forward in our understanding of biodiversity indices and soil properties as affected by different degrees of *Phragmites* density, and provides valuable insights in advancing knowledge regarding the influence of different degrees of *Phragmites* density on soil microbial biomass and mycorrhizal inoculum potential. However, further research must be undertaken, concerning more native and non-native *Phragmites* on a wide geographic scale to identify species-specific invasion mechanisms. This work must include allelopathic processes, in particular the soil systems that may lead to a better understanding of the mechanisms related to soil mutualistic community degradation and their associated effects in natural ecosystems

## Methods

### Study site

**Figure 7:**
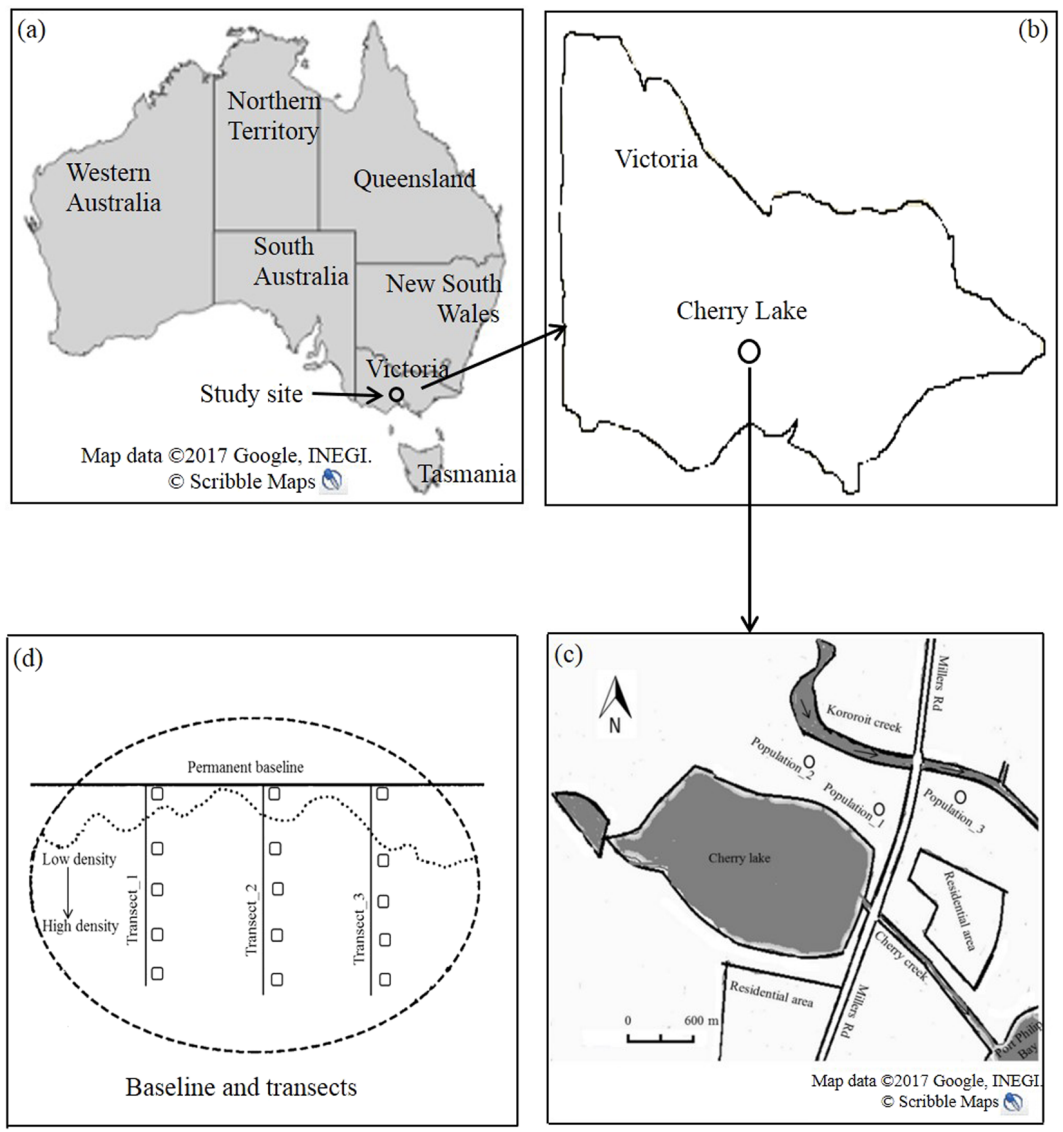
Study sites with a diagram illustrating basic design of quadrat sampling in Cherry Lake, Melbourne, Victoria, Australia. Maps created using Scribble Maps (basic version) with Google Maps: Maps of (**a**) Australia; retrieved from https://www.scribblemaps.com/create/#id=DSSy9BMlR0&lat=28.130127737873583&lng=36.680908203125&z=5&t=terrain; (**b**) Victoria (one of the states of Australia) and (**c**) study site (Cherry lake, Altona, Victoria, Australia); retrieved from https://www.scribblemaps.com/create/#id=DSSy9BMlR0&lat=37.86202452381126&lng=144.8261281708252&z=14&t=terrain; (**d**) Squares in transect and baseline diagram denote 1 × 1 m quadrats. Dotted line represents the edge of *Phragmites australis* population. Transects extended from low to high density into the area occupied by the *Phragmites australis*.

We conducted studies on the three natural stands of *Phragmites* population adjacent to Cherry lake (37°51′30″S, 144°50′5″E) (Fig. 7). An online tool called Scribble Maps (using the basic version) was used to create maps of (a) Australia; (b) Victoria (one of the states of Australia) and (c) study site (Cherry lake, Altona, Victoria, Australia)^49^. Scribble Maps is an advanced geographic information system and annotation tool that allows for the creation of custom maps^49^. Terrain views were used to identify target areas for the maps of the study sites (Fig. 7). Each population was separated from the others by a distance of at least 700 m. Cherry lake is a large remnant of a historically coastal wetland, Victoria, Australia.

### Diversity measurement

To evaluate the impact of invasion, field works were conducted mid-February 2014. A 75 m baseline was established through each *Phragmites* population, and 3 perpendicular transect lines, extending from low to high density into the area occupied by *Phragmites*, were chosen randomly along the baseline (Fig. 7d). The cover values of *Phragmites* were classified into five gradients, corresponding to zero (0%), slight (5~25%), moderate (26~50), high (51~75) and severe (76~100%)^50^. Alternatively, each transect was arranged into five density categories according to degrees of *Phragmites* density ranging from zero to severe level. Point-intercept sampling design along a transect line was used to collect soil and plant sample. Five (1 × 1-m) quadrats at 3 m intervals from baseline were established along a 12-m transect line with a density gradient of *Phragmites*. In total, 45 (3 × 5 × 3) vegetation quadrats from 3 populations were sampled for soil and plant diversity measurement. In each quadrat, all species of vascular plants and individual numbers of each species were recorded. Biological diversity was measured in terms of species richness (*S*), Shannon-Wiener diversity index (*H)*, species evenness (*E*) and Simpson’s reciprocal index (*1/D*) according to Keller^51^. Evenness was calculated as *H*/ln *S*, where *S* is the species richness expressed as the number of species.

### Laboratory analysis

Soil samples were collected along each transect, encompassing a gradient representing a range of *Phragmites* densities from absent to dense *Phragmites* growth. At 3 m intervals along the transect 1 m^2^ quadrats were made and three soil samples with 5 cm radius and 10 cm deep of *Phrgamites* rhizosphere from each density degree were collected. A total of 15 soil cores was taken from each transect i.e. in total, 135 (3 × 3 × 5 × 3) soil samples were collected from three populations. All soil samples were stored in sealed sterile bags and immediately transported back to the laboratory. The soil samples were passed through a 2 mm sieve to remove all debris. The three soil cores from each individual quadrat were thoroughly mixed to form a composite sample and then stored in a refrigerator at 4 °C for further processing. Soil samples were weighed prior to each further physical-chemical-microbial property analysis depending on the methods.

Soil water content (WC) was determined by sampling 5 g of composite soil using the gravimetric method^52^. Soil pH and electrical conductivity (EC) were determined with a pH meter ((Pocket digital pH meter, 99559, DickSmith Electronics, Australia) and conductivity meter (TPS Digital conductivity meter, 2100, TPS Pty Ltd., Australia) with a 1: 2.5 w/v (soil: distilled water) ratio^53^. Soil phenolics was determined by sampling 100 mg of air dried soil following the Folin-Ciocalteu method^54^. Soil organic matter (SOM) was measured by sampling 5 g of air dried soil using the loss on ignition method^55^.

Soil arbuscular mycorrhizal fungi (AMF) spores or sporocarps were extracted from 25 g air dried soil in triplicate for each sample by wet-sieving followed by flotation centrifugation in 50% sucrose^56^. The finest sieve was 53 µm. The spores were collected on a grid patterned (4 × 4 mm) filter paper, washed with distilled water to spread them evenly over the entire grid and counted using a dissecting microscope at ×30 magnification. A sporocarp was counted as one spore. The number of spores was expressed as the mean of three replicates. Soil dehydrogenase activity (DHA) was determined using the method of Gu *et al*.^57^ with little modification. Briefly, 2 g fresh sieved soil (2 mm) and 4 ml of 2 g L^−1^ 2, 3, 5-triphenyltetrazolium chloride (TTC) were mixed thoroughly using mixer (Vortex mixer, VM1, Ratek Instruments Pty Ltd., Victoria, Australia) and incubated at 37 °C in dark for 24 h. After that 0.5 ml of 1 M sulphuric acid was added to stop the reaction and centrifuged at 4 °C and 4000 rpm for 10 min with addition of 4 ml of ethyl acetate. The colour intensity of the supernatant was measured at 485 nm in spectrophotometer (Biochrom Libra S12, England) with ethyl acetate as a blank. The DHA has been used to measure overall biological activity in soil^58^.

Soil microbial biomass C, N and P were determined by chloroform fumigation–extraction method using adjusted (water holding capacity) moisturised soil due to reactivate the soil microbes^59–61^. The soil samples were incubated in the dark for two weeks at 25 °C. Two sets of samples (5 g of each fumigated and un-fumigated, soil samples were weighed separately into 50 ml centrifuge tubes and 20 ml of 0.5 M K_2_SO_4_ added to each. To one set of samples, 0.5 ml of ethanol free chloroform was added. Both fumigated and un-fumigated samples were capped and shaken simultaneously for 1 h. After shaking, the suspensions were allowed to settle for 10 min and the supernatants were filtered through Whatman No. 42 filter. For the sub-samples with chloroform, only the top 15 ml of the supernatant was filtered to reduce the amount chloroform in the filtrate. Filtrates from fumigated soil were also immediately bubbled with air for 30 min to remove any residual chloroform. Blanks were treated in the same manner. Then, soil microbial biomass C, N and P was determined using the filtrates accordingly.

Total Organic Carbon (TOC) was determined following the procedures of Wüthrich *et al*.^62^ with TOC analyser (SHIMADZU TOC-5000A). The difference in TOC between the fumigated and non-fumigated samples corresponds to the microbial carbon in the soil. TOC-contents were divided by 0.33 (*K*_EC_) to estimate the C content of the microbial biomass^60^. Total N was determined using the method of Cabrera and Beare^61^ with ion chromatograph (Shimadzu Ion Chromotograph, Kyoto, Japan) and calculated using *K*_EN_-factor (0.54)^63^. Soil inorganic P was extracted with 0.5 M NaHCO_3_ and measured photometrically (UV/Visible spectrophotometer, Biochrom Libra S12, England) at 882 nm as a blue phosphate molybdic acid complex^64^ and microbial P was calculated using *K*_EP_-factor (0.4)^64^.

### Mycorrhizal inoculum potential bioassay

Each pot containing 100 g sieved composite soil from *Phragmites* density gradient soil was moistened uniformly with distilled water and incubated for 48 hrs at 25 °C. *Melaleuca ericifolia* seeds were selected for bioassay as this plant is a native wetland shrub and not commonly found in fresh and brackish water swamps across south-eastern Australia. The seeds were surface sterilised by washing in 70% ethyl alcohol for duration of 10 minutes. The seeds were sown on the soil surface in 45 mm plastic pots. Pots were placed in growth chamber (Westinghouse, Electrolux home products, Australia) set to 25/15 °C day/night temperature and a 12 h photoperiod with illumination of 84 µmols^−1^m^−2^. After 2 weeks, the seedlings were thinned in equal number (10 per dish) for growth and establishment. After three months, plant biomass was measured and the sampling of root tissue was undertaken by obtaining the roots of five plants for each treatment including the control. The staining of the roots for determining AMF root colonisation was completed accordingly^65, 66^. Stained root sections were then examined with light microscopy through detecting either the presence or absence of arbuscules or hypha in sample root tissue^67^.

### Statistical analyses

The impact of *Phragmites* density on individual species was assessed by analysing changes in other species density among the density categories (zero to severe level) in each population. The ‘Kruskal–Wallis’ test, followed with pairwise comparisons using the Dunn-Bonferroni approach was adopted, as the data did not satisfy assumptions of normality and homogeneity of variance. One-way ANOVA with Bonferroni post-hoc analysis was conducted to compare biodiversity indices among different populations. Regression analysis was conducted to test the responsibility of *Phragmites* density to cause the variation in the diversity indices and soil properties. Again, one-way ANOVA with Bonferroni post-hoc analysis was conducted separately for each population to compare physico-chemical-biological variables among density categories. The Spearman correlation was used to assess the relationships between soil properties and biodiversity indices. The relationships among components of diversity and soil properties were quantified with correlation and principal components analyses (PCA). Principal components were based on correlation matrices, and were interpreted and presented if eigenvalues were ≥1^68^. Principal-components analyses were conducted individually for each of the three populations. AMF colonization and biomass data from the AMF inoculum potential of soil experiment were subjected to one-way and two-way ANOVA with significant test by LSD.

## References

[1] Mack RN (2000). Biotic invasions: causes, epidemiology, global consequences, and control. Ecol. Appl..

[2] Alvarez ME, Cushman JH (2002). Community-level consequences of a plant invasion: effects on three habitats in coastal California. Ecol. Appl..

[3] Gordon DR (1998). Effects of invasive, non‐indigenous plant species on ecosystem processes: lessons from Florida. Ecol. Appl..

[4] Callaway RM, Thelen GC, Rodriguez A, Holben WE (2004). Soil biota and exotic plant invasion. Nature.

[5] Pyšek P (2003). Vegetative regeneration in invasive Reynoutria (Polygonaceae) taxa: the determinant of invasibility at the genotype level. Am. J. Bot..

[6] Loreau M (2001). Biodiversity and ecosystem functioning: current knowledge and future challenges. Science.

[7] Comín FA, Romero JA, Hernández O, Menéndez M (2001). Restoration of wetlands from abandoned rice fields for nutrient removal, and biological community and landscape diversity. Restor. Ecol..

[8] Zedler JB, Kercher S (2004). Causes and consequences of invasive plants in wetlands: opportunities, opportunists, and outcomes. Crit. Rev. Plant Sci..

[9] Uddin MN, Caridi D, Robinson RW (2012). Phytotoxic evaluation of *Phragmites australis*: an investigation of aqueous extracts of different organs. Mar. Freshwater Res..

[10] Plut K, Paul J, Ciotir C, Major M, Freeland JR (2011). Origin of non-native *Phragmites australis* in North America, a common wetland invader. Fundam. Appl. Limnol..

[11] Uddin MN, Robinson RW, Caridi D, Al Harun MAY (2014). Suppression of native Melaleuca ericifolia by the invasive *Phragmites australis* through allelopathic root exudates. Am. J. Bot..

[12] Meyerson, L. A., Cronin, J. T. & Pyšek, P. *Phragmites australis* as a model organism for studying plant invasions. *Biol. Invasions* 1–11 (2016).

[13] McCormick MK, Kettenring KM, Baron HM, Whigham DF (2010). Extent and reproductive mechanisms of *Phragmites australis* spread in brackish wetlands in Chesapeake Bay, Maryland (USA). Wetlands.

[14] Uddin MN, Robinson RW, Buultjens A, Al Harun MAY, Shampa SH (2017). Role of allelopathy of *Phragmites australis* in its invasion processes. J. Exp. Mar. Biol. Ecol..

[15] Meyerson LA, Saltonstall K, Windham L, Kiviat E, Findlay S (2000). A comparison of Phragmites australis in freshwater and brackish marsh environments in North America. Wetlands Ecol. Manage..

[16] Mozdzer TJ, Zieman JC (2010). Ecophysiological differences between genetic lineages facilitate the invasion of non‐native *Phragmites australis* in North American Atlantic coast wetlands. J. Ecol..

[17] Ehrenfeld JG (2003). Effects of exotic plant invasions on soil nutrient cycling processes. Ecosystems.

[18] Kourtev PS, Ehrenfeld JG, Häggblom M (2002). Exotic plant species alter the microbial community structure and function in the soil. Ecology.

[19] Ravit B, Ehrenfeld JG, Haggblom MM (2003). A comparison of sediment microbial communities associated with *Phragmites australis* and *Spartina alterniflora* in two brackish wetlands of New Jersey. Estuaries.

[20] Bodelier P, Libochant JA, Blom C, Laanbroek HJ (1996). Dynamics of nitrification and denitrification in root-oxygenated sediments and adaptation of ammonia-oxidizing bacteria to low-oxygen or anoxic habitats. Appl. Environ. Microbiol..

[21] Allison SD, Nielsen C, Hughes RF (2006). Elevated enzyme activities in soils under the invasive nitrogen-fixing tree *Falcataria moluccana*. Soil Biol. Biochem..

[22] Broz AK, Manter DK, Vivanco JM (2007). Soil fungal abundance and diversity: another victim of the invasive plant Centaurea maculosa. The ISME journal.

[23] McCarthy M, Pratum T, Hedges J, Benner R (1997). Chemical composition of dissolved organic nitrogen in the ocean. Nature.

[24] Parker IM (1999). Impact: toward a framework for understanding the ecological effects of invaders. Biol. Invasions.

[25] Lachavanne, J.-B. & Juge, R. *Biodiversity in land-inland water ecotones*. Vol. 18 (Taylor & Francis, 1997).

[26] Li L (2014). A Comparison of the functional traits of common reed (*Phragmites australis*) in northern china: aquatic vs. terrestrial ecotypes. PLoS ONE.

[27] Daehler CC (2003). Performance comparisons of co-occurring native and alien invasive plants: implications for conservation and restoration. Annu. Rev. Ecol. Evol. Syst..

[28] Sax DF, Gaines SD (2003). Species diversity: from global decreases to local increases. Trends Ecol. Evol..

[29] Qi S-S (2014). Curvilinear effects of invasive plants on plant diversity: plant community invaded by *Sphagneticola trilobata*. PLoS ONE.

[30] Rodriguez LF (2006). Can invasive species facilitate native species? Evidence of how, when, and why these impacts occur. Biol. Invasions.

[31] White LF, Shurin JB (2011). Density dependent effects of an exotic marine macroalga on native community diversity. J. Exp. Mar. Biol. Ecol..

[32] Melo FP, Arroyo-Rodríguez V, Fahrig L, Martínez-Ramos M, Tabarelli M (2013). On the hope for biodiversity-friendly tropical landscapes. Trends Ecol. Evol..

[33] Haslam, S. Community regulation in Phragmites communis Trin.: I. monodominant stands. *J. Ecol*. 65–73 (1971).

[34] Rudrappa T (2009). Phragmites australis root secreted phytotoxin undergoes photo-degradation to execute severe phytotoxicity. Plant Signal. Behav..

[35] Zhou X, Wu F (2012). p-coumaric acid influenced cucumber rhizosphere soil microbial communities and the growth of *Fusarium oxysporum* f. sp. cucumerinum owen. PLoS ONE.

[36] Saviozzi A, Levi-Minzi R, Cardelli R, Riffaldi R (2001). A comparison of soil quality in adjacent cultivated, forest and native grassland soils. Plant Soil.

[37] Stefanowicz AM, Stanek M, Nobis M, Zubek S (2016). Species-specific effects of plant invasions on activity, biomass, and composition of soil microbial communities. Biol. Fertility Soils.

[38] Martina JP, Hamilton SK, Turetsky MR, Phillippo CJ (2014). Organic matter stocks increase with degree of invasion in temperate inland wetlands. Plant Soil.

[39] McIntosh, P., Loeseke, M. & Bechler, K. Soil changes under mouse-ear hawkweed (*Hieracium pilosella*). *N. Z. J. Ecol*. 29–34 (1995).

[40] Yang N, Zou D, Yang M, Lin Z (2016). Variations in soil microbial biomass carbon and soil dissolved organic carbon in the re-vegetation of hilly slopes with purple soil. PLoS ONE.

[41] Sanon A (2009). Changes in soil diversity and global activities following invasions of the exotic invasive plant, *Amaranthus viridis* L., decrease the growth of native sahelian Acacia species. FEMS Microbiol. Ecol..

[42] Bradley K, Drijber RA, Knops J (2006). Increased N availability in grassland soils modifies their microbial communities and decreases the abundance of arbuscular mycorrhizal fungi. Soil Biol. Biochem..

[43] Yang W, Jeelani N, Leng X, Cheng X, An S (2016). *Spartina alterniflora* invasion alters soil microbial community composition and microbial respiration following invasion chronosequence in a coastal wetland of China. Sci. Rep..

[44] Roberts KJ, Anderson RC (2001). Effect of garlic mustard [*Alliaria Petiolata* (Beib. Cavara & Grande)] extracts on plants and arbuscular mycorrhizal (AM) Fungi. Am. Midl. Nat..

[45] Stinson KA (2006). Invasive plant suppresses the growth of native tree seedlings by disrupting belowground mutualisms. PLoS Biol.

[46] Uddin MN, Robinson RW, Caridi D, Harun MA (2014). Is phytotoxicity of *Phragmites australis* residue influenced by decomposition condition, time and density?. Mar. Freshwater Res..

[47] Uddin MN, Robinson RW, Caridi D (2014). Phytotoxicity induced by *Phragmites australis*: An assessment of phenotypic and physiological parameters involved in germination process and growth of receptor plant. J. Plant Interact..

[48] Rudrappa T, Bonsall J, Gallagher JL, Seliskar DM, Bais HP (2007). Root-secreted allelochemical in the noxious weed *Phragmites Australis* deploys a reactive oxygen species response and microtubule assembly disruption to execute rhizotoxicity. J. Chem. Ecol..

[49] Scribble Maps. Retrieved from https://www.scribblemaps.com/ (2017).

[50] Sinkins PA, Otfinowski R (2012). Invasion or retreat? The fate of exotic invaders on the northern prairies, 40 years after cattle grazing. Plant Ecol..

[51] Keller BE (2000). Plant diversity in *Lythrum*, *Phragmites*, and *Typhamarshes*, Massachusetts, USA. Wetlands Ecol. Manage..

[52] Little K, Metelerkamp B, Smith C (1998). A comparison of three methods of soil water content determination. S.Afr. J. Plant Soil.

[53] Paz‐Ferreiro J, Trasar‐Cepeda C, Leirós M, Seoane S, Gil‐Sotres F (2007). Biochemical properties of acid soils under native grassland in a temperate humid zone. N. Z. J. Agric. Res..

[54] Blainski A, Lopes GC, De Mello JCP (2013). Application and analysis of the Folin Ciocalteu method for the determination of the total phenolic content from *Limonium Brasiliense* L. Molecules.

[55] Beaudoin A (2003). A comparison of two methods for estimating the organic content of sediments. J. Paleolimnol..

[56] Wang F-Y, Liu R-J, Lin X-G, Zhou J-M (2004). Arbuscular mycorrhizal status of wild plants in saline-alkaline soils of the Yellow River Delta. Mycorrhiza.

[57] Gu Y, Wang P, Kong C (2009). Urease, invertase, dehydrogenase and polyphenoloxidase activities in paddy soil influenced by allelopathic rice variety. Eur. J. Soil Biol..

[58] McLatchey GP, Reddy KR (1998). Regulation of organic matter decomposition and nutrient release in a wetland soil. J. Environ. Qual..

[59] Brookes PC, Kragt JF, Powlson DS, Jenkinson DS (1985). Chloroform fumigation and the release of soil nitrogen: The effects of fumigation time and temperature. Soil Biol. Biochem..

[60] Sparling GP, West AW (1988). A direct extraction method to estimate soil microbial C: calibration *in situ* using microbial respiration and 14C labelled cells. Soil Biol. Biochem..

[61] Cabrera M, Beare M (1993). Alkaline persulfate oxidation for determining total nitrogen in microbial biomass extracts. Soil Sci. Soc. Am. J..

[62] Wüthrich C, Schaub D, Weber M, Marxer P, Conedera M (2002). Soil respiration and soil microbial biomass after fire in a sweet chestnut forest in southern Switzerland. CATENA.

[63] Brookes PC, Landman A, Pruden G, Jenkinson D (1985). Chloroform fumigation and the release of soil nitrogen: a rapid direct extraction method to measure microbial biomass nitrogen in soil. Soil Biol. Biochem..

[64] Brookes P, Powlson D, Jenkinson D (1982). Measurement of microbial biomass phosphorus in soil. Soil Biol. Biochem..

[65] Bainard LD, Klironomos JN, Hart MM (2010). Differential effect of sample preservation methods on plant and arbuscular mycorrhizal fungal DNA. J. Microbiol. Meth..

[66] Moorman, T. & Reeves, F. B. The role of endomycorrhizae in revegetation practices in the semi-arid west. II. A bioassay to determine the effect of land disturbance on endomycorrhizal populations. *Am. J. Bot*. 14–18 (1979).

[67] Burke DJ, Hamerlynck EP, Hahn D (2002). Effect of arbuscular mycorrhizae on soil microbial populations and associated plant performance of the salt marsh grass Spartina patens. Plant Soil.

[68] Cliff N (1988). The eigenvalues-greater-than-one rule and the reliability of components. Psychol. Bull..

